# *β*-Lactoglobulin Influences Human Immunity and Promotes Cell Proliferation

**DOI:** 10.1155/2016/7123587

**Published:** 2016-11-13

**Authors:** Chun San Tai, Yi Yun Chen, Wen Liang Chen

**Affiliations:** ^1^Department and College of Biological Science and Technology, National Chiao Tung University, Hsinchu, Taiwan; ^2^Institute of Molecular Medicine and Bioengineering, National Chiao Tung University, Hsinchu, Taiwan

## Abstract

*β*-Lactoglobulin (LG) is suspected to enhance or modulate human immune responses. Moreover, LG is also hypothesized to increase human cell proliferation. However, these potential functions of LG have not been directly or thoroughly addressed. In this study, we demonstrated that LG is a potent stimulator of cell proliferation using a hybridoma cell (a splenocyte fused with a myeloma cell) model. LG's ability to promote cell proliferation was lost when the protein is denatured. To further investigate the influence of LG's conformation on cell proliferation, we chemically modified LG by either carboxymethylation (CM) or acetylation and observed significantly reduced cell proliferation when the protein structure was altered. Furthermore, we proved that LG enhances cell proliferation via receptor-mediated membrane IgM receptor. These data indicated that nondenatured LG is the major component in milk that modulates cell proliferation. Collectively, our study showed that LG plays a key role in enhancing immune responses by promoting cell proliferation through IgM receptor.

## 1. Introduction

*β*-Lactoglobulin (LG) is the most abundant whey protein in bovine milk. Notably, LG accounts for more than 50% of total whey protein [1]. LG has been widely studied in the food industry because of its nutritional and functional effects on various biological processes [2]. The actual biological function of LG has not been studied profoundly [3–5]. Based on the structure of LG, it is thought to bind ligands and transport small hydrophobic ligands [6]. Because LG has multiple ligand binding sites, LG is capable of binding to vitamins A and D, palmitic acid, and other hydrophobic compounds. Its calyx structure forms a central hydrophobic cavity that binds to these molecules [5, 7–11]. In addition, LG exhibits strong binding affinities for fatty acids, phospholipids, and aromatics compounds. Binding of these molecules to LG can alter their biological activities. Examples of this include angiotensin-converting enzyme (ACE) inhibition, alterations in antimicrobial and anticarcinogenic activity, hypocholesterolemic and metabolic effects, and modulation of other physiological functions [12]. Due to its ability to bind hydrophobic molecules, LG was used to improve the encapsulation properties of liposomes and to serve as a stable system for vitamin E delivery [13].

In addition to its ligand binding ability, LG also contributes to the defense against severe diseases. Sepsis and septic shock, which are often caused by bacterial infection, are systematic inflammatory responses that induce multiorgan failure and results in a high mortality rate in critically ill patients [14, 15]. The guidelines of treating sepsis and septic shock are to perform cardiorespiratory resuscitation and arrest uncontrolled infections [16]. To effectively mitigate infections and rescue organ dysfunctions, current strategies often combine antimicrobial therapy to suppress further infections and antioxidant treatment to control oxidative stress within the mitochondria [16, 17]. The combination of antimicrobial therapy and antioxidant treatment is an effective and promising treatment for sepsis and septic shock [18, 19]. Numerous studies indicate that LG possesses extra functions which include antimicrobial and antioxidant activities [20, 21]. Chaneton et al. identified LG's antimicrobial activity while studying bacterial infection of the mammary glands [22].

Additionally, LG may be an inexpensive antioxidant nutrient that is easily and readily accessible. These are appealing traits because antioxidant depletion, nitric oxide overproduction, mitochondrial dysfunction, and decreased ATP concentrations are often observed in septic patients and antioxidant compounds have been proven to reduce the mortality rate of critically ill patients [23]. LG may potentially act as an antioxidant nutrient that is easily accessible and cheap in daily life. Our previous report showed that the free cysteine of LG plays a protective role in the antioxidant nature of milk. LG is responsible for 50% of milk's antioxidant activity [24]. Not only can LG act directly as an antioxidant nutrient, it can also carry other antioxidants via its ligand binding pocket. Thus, it increases both the bioavailability and amount of available antioxidants.

Curcumin showed strong antioxidant and anti-inflammatory effects in an experimental rat model of sepsis. However, curcumin is poorly absorbed and rapidly metabolized. The addition of LG was reported to overcome these major limitations of curcumin. LG binds to curcumin; thus, it enhances its bioactivity and improves its antioxidant ability [25, 26]. LG has various biological functions that could potentially improve our health and enhances our immune responses. The purpose of this study was to investigate the other potential effects of LG on the immune system.

Some earlier studies have examined LG's ability to affect immune responses. For example, LG mediates thymic stromal lymphopoietin through the NF-kB pathway which demonstrates its functional role in the immune system [27]. Whey protein, which is approximately 20% of the total protein found in milk, affects diverse biological processes ranging from bone growth, wound healing, immune regulation, control of infections, and metabolism [28]. Growing evidences directly show the functional role of whey protein in immune enhancement and modulation; these studies have used both tissue culture systems and animal models. In animal models, whey protein is reported to improve mucosal immunity and innate cells during suckling [29]; further, it enhances immune cell proliferation and migration to secondary lymphoid organs [30]. Furthermore, Belford et al. have demonstrated that bovine whey is a potent source of growth-promoting activity for all mesodermal-derived cells tested, including human skin, human embryonic lung fibroblasts, Balb/c 3T3 fibroblasts, and rat L6 myoblasts [31]. Bovine milk and bovine whey proteins has also been tested as a serum substitutes. Pakkanen et al. and Capiaumont et al. utilized bovine milk and bovine whey proteins to culture hybridoma cells and they found that whey proteins were the most important ingredients in milk that stimulated cell proliferation [32, 33]. Taking all together, whey protein exhibits several benefits. It not only provides nutrition, but also enhances immune activities and promotes cell proliferation. However, the mechanisms by which whey protein or any specific part of whey protein contributes immune modulation have not yet been elucidated. Whey protein is comprised of several immune enhancing components, including lactoferrin and LG, which can modulate immune responses [1, 34, 35]. In this study, we aimed to use hybridoma cells as a model to investigate the functional role of LG as a stimulator of cell proliferation. In the following sections, we demonstrate that LG is a major protein in milk that enhances cell proliferation. Further, we showed that when LG is thermally denatured, acetylated, or carboxymethylated it loses a substantial amount of its activity. The LG receptor was isolated and identified as a membranous IgM using a LG affinity column and MALDI-TOF MS. These findings suggest that LG is the critical component of whey protein that induces cell proliferation via membranous IgM. The physiological significance of these findings is also presented and discussed.

## 2. Materials and Methods

### 2.1. Materials

Freshly bulked whole raw milk was obtained from a Chiayi local dairy farm and was immediately centrifuged at 13,000 rpm (15,500 ×g) for 1 hour at 4°C. Then, the supernatant was carefully removed and the remaining fraction (whey protein) was saturated with 30% ammonium sulfate. LG was purified from the top fraction using a G-150 chromatography column as described previously [5, 36, 37]. HLG is LG that was heated at 95°C for 5 min. All chemicals were from Sigma and columns were from GE Healthcare Life Sciences.

### 2.2. Acetylation and Carboxymethylation of LG

LG was acetylated using a previously described method [8, 37]. Briefly, LG (2 mL) in aqueous sodium bicarbonate (50 mM, pH 8.0) containing urea (6 M) and acetic anhydride (5 *μ*L) was slowly added into the reaction mixture, while the pH was maintained at 8.0 using NaOH (0.1 M). After a 3-hour incubation at approximately 25°C, the acetylated protein was desalted on a column (Bio-Gel P2), eluted with ammonium bicarbonate (0.05 M), and lyophilized.

For carboxymethylation, LG (5 mg) was first dissolved in Tris-HCl buffer (5 mL, 0.1 M, pH 8.6) containing ultra-pure urea (6 M) and 2-mercaptoethanol (0.02 M) [8, 37, 38]. After flushing the tube with nitrogen, iodoacetic acid (20 mg) was added to the reaction mixture, while the pH of the reaction mixture was maintained at 8.6 by adding NaOH (0.1 M). Then, the sample was incubated for 3 hours. Carboxymethylated LG (CM-LG) was desalted on a column (Bio-Gel P2), eluted with ammonium bicarbonate (0.05 M), and lyophilized. According to amino acids analysis, the CM-LG contained 4.9 residues of CM-cysteine per mole of LG.

### 2.3. Preparation of the LG Affinity Column

LG (100 mg) was dissolved and dialyzed in a coupling buffer containing 0.1 M NaHCO_3_ and 0.5 M NaCl at pH 8.3. Crude LG was then coupled with CNBr-activated Sepharose-4B (Pharmacia, Uppsala, Sweden) according to the manufacturer's instructions with some modifications [39]. Briefly, 3.33 g of freeze-dried Sepharose was resuspended in 20 mL of 1 mM HCl and then immediately washed with 20x volume of the same solution. Within 15 min, the sample was applied on a glass filter [39]. The gel was then washed with the coupling buffer (0.1 M NaHCO_3_, 0.5 M NaCl, pH 8.3) and degassed. LG was added to the gel (in 15 mL), while gently stirring the solution using a magnetic bar for 1 hour. After coupling, the gel was washed with 10x volume of PBS to remove unbound materials via a sintered glass filter. The gel was then treated with a blocking solution (0.1 M Tris-HCl, 0.5 M NaCl, pH 8.0) for 2 hours to saturate the remaining reactive sites. The degassed gel was then washed with 3 cycles of blocking buffer and a 0.15 M NaCl solution at pH 11.0 (adjusted by ammonium). Finally, the gel was equilibrated in PBS and packed onto a 1.5 × 20 cm column.

### 2.4. Cell Culture

In order to continue and compare our results with previous investigations [36], we used murine hybridoma cells against prostatic-specific antigen (a splenocyte fused with a myeloma cell). Cells were cultured in Dulbecco's Modified Eagle's Medium (GIBCO, Gaithersburg, MD) containing L-glutamine (10%, Boehringer, M12-702, Mannheim, Germany), bovine calf serum (10%, Jacques Boy, Reims, France), hypoxanthine thymidine supplement (10%, GIBCO), penicillin (100 U/mL, GIBCO), and streptomycin (100 U/mL, GIBCO) at 37°C in a CO_2_ (5%) atmosphere at 99% humidity. Cells in the exponential phase of growth, from cultures with a minimum density 1 × 10^6^ cells/mL, were collected and used for experiments. The culture medium was changed every two days.

### 2.5. MTT Assay for the Evaluation of Cell Proliferation Effect

Cells were seeded at a density 1 × 10^4^ cells/coated well, treated with either LG, CM-LG, acetylated LG, or HLG, and incubated at 37°C in a humidified atmosphere (5% CO_2_). After predetermined periods of time, cell layers were rinsed with PBS and a 3-(4, 5-dimethylthiazol-2-yl)-2,5-diphenyl tetrazolium bromide (MTT) solution (50 *μ*L, 12 mM) was added to each well. After 4 hours at 37°C, the MTT solution was removed and the insoluble formazan crystals that formed were dissolved in dimethyl sulfoxide (DMSO, 150 *μ*L). The absorbance of the formazan product was measured at 540 nm using a spectrophotometer (Molecular Devices, Sunnyvale, California) [40, 41].

### 2.6. Preparation of FITC-LG Conjugation

LG (Sigma, St. Louis, MO, USA) was dissolved in aqueous sodium bicarbonate (0.1 M, pH 9.6). Fluorescein isothiocyanate (FITC; Sigma, St. Louis, MO, USA) was dissolved in DMSO. The LG (1800 *μ*L) and FITC solutions (200 *μ*L) were mixed and incubated for 90 min at approximately 23°C in the dark with constant stirring. The FITC-LG conjugate was separated from free FITC by dialysis in PBS (pH 7.4, Pharmacia, Sweden).

### 2.7. Flow Cytometry

Samples (1 × 10^7^ cells) were incubated with FITC-LG at various concentrations (0, 0.0625, 0.125, 0.25, 0.5, and 1 mg) at a designated temperature for 30 min and then evaluated by flow cytometry. We used a flow cytometer (XL, Coultronics-Margency, France) equipped with an argon laser set at 488 nm and the green fluorescence was processed with a filter (bandpass 520–530 nm). Fluorescence was displayed as a monoparametric histogram (256 channels, logarithmic scale) and expressed as the mean intensity of fluorescence (MIF): MIF = *e* ^[(ln⁡1000:256)*x*]^; *x* is the mean peak channel on a logarithmic scale. For each assay, 1 × 10^4^ cells were analyzed. Viable cells were selected using the biparametric histogram FLS × 90LS (size × granularity).

### 2.8. Confocal Microscopy

Samples (1 × 10^7^ cells) were incubated with FITC-LG (1 mg) at the designated temperature for 30 min. The cells were centrifuged and washed with PBS (pH 7.4) three times. Samples were fixed with paraformaldehyde (4%) for 30 min and washed with PBS (pH 7.4). Samples were then examined using a confocal microscope; optical sections were obtained with an epifluorescence inverted microscope (Olympus IX-70) equipped with a cell scanner (EPR™ system, Scanalytics, Billerica, MA) and a water immersion apochromatic lens (60x PSF: 1.2-NA, Olympus, France). Scanning along the optical axis was performed with a piezoelectric *z*-axis focus device (*z* spacing 0.25 mm). Images were collected on a cooled charge-coupled device camera (12 bits, Princeton Instruments, USA). With a filter set (WIB cube, Olympus), we selected the fluorescence excitation (460–490 nm) and the integral part of the emission spectrum (BA515). An image intensity-calibration kit (InSpeck, Molecular Probes, Eugene City, OR) was used for calibration. A blank image of the detector dark current and the background were removed from each image acquired pixel by pixel.

### 2.9. LG Receptor Purification

Plasma membrane-enriched preparations were obtained from the cultured hybridoma cells. Briefly, approximately 1 × 10^6^ cells/mL were disrupted in ice-cold PBS (pH 7.4) containing 1% PMSF and 1% Tween-20. The homogenate was sonicated to facilitate the release of membrane proteins and then centrifuged (5 min at 10000 ×g). The supernatant was collected, loaded onto the LG affinity column, and incubated for 1 hour. Proteins were eluted from the column using a 100 mM glycine buffer (pH 3) and fractions were collected. The fractions were neutralized using 50 *μ*L of 1 M Tris (pH 11) per 1 mL volume of fraction. The absorbance at 280 nm for each fraction was measured to determine which ones contained the eluted LG receptor. Protein rich samples were analyzed by matrix-assisted laser desorption/ionization time-of-flight mass spectrometry (MALDI-TOF MS) to identify the LG receptor.

### 2.10. MALDI-TOF MS

Protein spots were excised manually from Coomassie blue stained gels and washed twice in 25 nM NH_4_HCO_3_ for 10 min until the gel pieces became transparent. Gel pieces were dehydrated with 100% acetonitrile for 5 min, dried, and incubated with 15 *μ*L of a 12.5 ng/*μ*L trypsin solution at 37°C overnight. Trypsinized samples were then concentrated using a C18 Zip-Tips Kit (Millipore) and eluted with 50% acetonitrile/0.1% trifluoroacetic acid according to the manufacturer's instructions. MALDI-TOF MS was performed using a Microflex MALDI-TOF LRF20 mass spectrometer (Bruker Daltonics, Billerica, MA). The Zip-Tip purified peptide digests were mixed with a *α*-cyano-4-hydroxycinnamic acid matrix and assessed in the reflection positive-ion mode using an accelerating voltage of 25 kV. Subsequently, proteins were identified using the MASCOT search engine from the SWISS-PROT database (http://www.matrixscience.com/).

### 2.11. Gel Electrophoresis

A 15% sodium dodecyl sulfate polyacrylamide gel (SDS-PAGE) was used to characterize the receptor. Our protocol was modified from [37] and similar to that described in [4, 42]. For the SDS-PAGE, all the fraction samples from the LG affinity column (5 *μ*g) were mixed with loading buffer (12 mM Tris-HCl, pH 6.8, 5% glycerol v/v, and 0.02% bromophenol blue m/v) with or without 2-mercaptoethanol (*β*-ME) (2.88 mM) and run for approximately 1.5 hours at 120 V.

### 2.12. Western Blotting

The gel from the SDS-PAGE was soaked instantly and briefly in a transfer buffer (25 mM Tris-HCl, 192 mM glycine, 20% methanol (v/v), and 0.0375% SDS (pH 8.3)) for 30 s [4, 36]. Then, the gel was immediately electrotransferred to a nitrocellulose membrane (Hybond-ECL extra, Amersham, Buckingham, UK) at 90 mA for 1 hour using a semidry transfer cell (BioRad, Hercules, CA). The membrane was immersed in 1% gelatin for 1 hour with gentle shaking. After 3 washes with PBS for 5 min each, the membrane was incubated with 1 mg of LG in a buffer (PBS with 0.5% gelatin and 0.05% Tween-20) for 1 hour. After 3 washes, the blot was incubated with mouse anti-LG conjugated to horseradish peroxidase (anti-LG-HRP) for 1 hour. Finally, the membrane was developed with 0.1 mg/mL of 3-3′-diaminobenzidine (3,3′,4,4′-tetra-amino-biphenyl) containing 0.01% H_2_O_2_ in PBS.

### 2.13. Statistical Analysis

Data are presented as the mean ± SD The differences among the groups were calculated using either a two-tailed Student's *t*-test or a one-way ANOVA test. A *p*-value of <0.05 indicated a statistically significant difference. The coefficients of variations among inter- and intra-assays were calculated by SD/mean × 100%.

## 3. Result

### 3.1. LG Regulated Cell Proliferation

**Figure 1:**
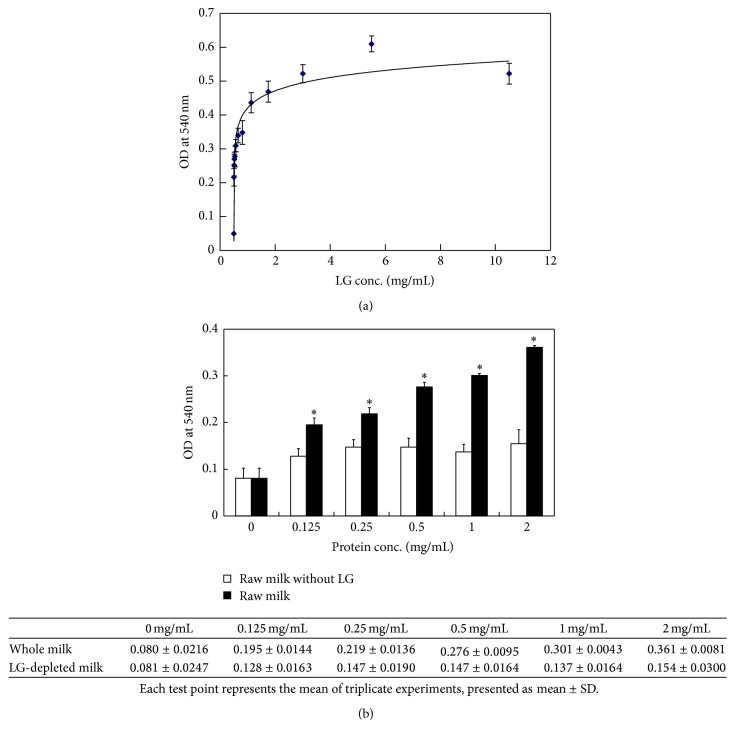
LG regulates cell proliferation. (a) Hybridoma cells were treated with different doses of LG for 72 hours. The MTT assay revealed that LG could stimulate cell proliferation in a dose-dependent manner. However, LG at a dose of 5 mg/mL achieved the maximal effect (OD at 540 nm = 0.6 ± 0.023). (b) Hybridoma cells were treated with different doses of whole or LG-depleted milk for 72 hours. Cell proliferation was significantly decreased by the LG-depleted milk treatment when compared with the whole milk treatment (^*∗*^ *p* < 0.001, two-tailed Student's *t*-test). Each experiment was performed in triplicate.

In this study, we first demonstrated the effect of LG on cell proliferation using a hybridoma cell model. As shown in Figure 1, the results of the MTT assay revealed a positive correlation between LG treatment doses and the extent of cell proliferation. The maximal effect on cell growth was reached using a LG treatment dose of 5 mg/mL (OD at 540 nm: 0.6 ± 0.023) (Figure 1(a)). To confirm that LG is the major protein in bovine milk that stimulates cell proliferation, we used an anti-LG affinity column to remove LG from bovine milk and treated the hybridoma cells with whole milk or LG-depleted milk. The results showed that the stimulation of cell proliferation was remarkably decreased when cells were treated with LG-depleted milk when compared to whole milk (OD at 540 nm: 0.36 for whole milk versus 0.15 for LG-depleted milk, *p* < 0.001) (Figure 1(b)).

### 3.2. Effect of Altered LG Conformation on Cell Proliferation

**Figure 2:**
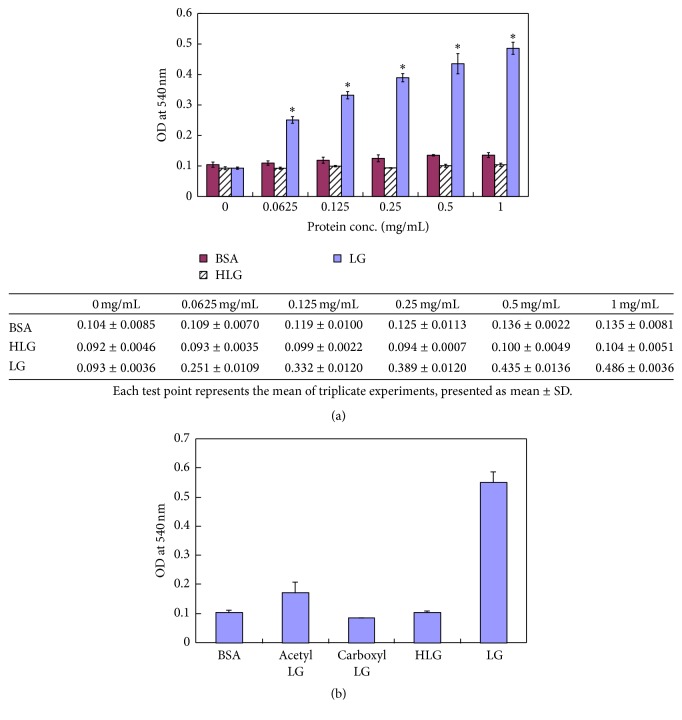
Effect of conformationally altered LG on cell proliferation. (a) Hybridoma cells were treated with different doses (0, 0.0625, 0.125, 0.25, 0.5, and 1 mg/mL) of LG, heated LG (HLG), and BSA (as baseline treatment for comparison), respectively, for 72 hours and cell proliferation was evaluated using the MTT assay (OD at 540 nm). Each experiment was performed in triplicate. When compared to the HLG and BSA treated cells, cell proliferation was remarkably induced by LG treatments of various doses (from 0.0625 to 1 mg/mL, *p* < 0.001 for each dose). The data from the MTT assay are shown in table. Note:  ^*∗*^ *p* value < 0.001 using a one-way ANOVA test. (b) To assess the effect of LG's conformation on its ability to stimulate cell proliferation, hybridoma cells were treated with normal LG (1 mg/mL), thermally denatured LG (HLG) (1 mg/mL), acetylated LG (1 mg/mL), and carboxymethylated LG (CM-LG) (1 mg/mL) for 72 hours. The MTT assay showed that only normal LG could significantly promote cell proliferation, whereas thermally denatured or chemically modified LGs had no such effect when compared to the BSA baseline treatment. (OD at 540 nm: 0.104 ± 0.008, 0.172 ± 0.035, 0.085 ± 0.002, 0.104 ± 0.005, and 0.552 ± 0.035 for BSA, acetyl LG, CM-LG, HLG, and normal LG, resp.; normal LG compared to BSA, acetyl LG, CM-LG, and HLG, *p* < 0.0001).

After confirming that LG enhanced cell proliferation, we further studied whether the conformation of LG was important for LG's ability to stimulate cell growth. In contrast to nondenatured LG, thermally denatured LG (HLG: LG was heated at 95°C for 5 min) did not show such great cell growth-promoting activity when compared to the BSA baseline treatment (OD at 540 nm: 0.104 ± 0.005 for HLG versus 0.552 ± 0.0355 for LG, *p* < 0.001) (Figure 2(a)). This result indicates that thermally denatured LG lost the ability to promote cell growth. Moreover, we used the carboxymethylated or acetylated LG to treat hybridoma cells and found that the cell growth-promoting effect of these chemically denatured LGs was markedly impaired. The ODs at 540 nm are 0.104 ± 0.008, 0.172 ± 0.035, 0.085 ± 0.002, 0.104 ± 0.005, and 0.552 ± 0.035 for BSA, acetyl LG, CM-LG, heated LG, and normal LG, respectively (*p* < 0.0001) (Figure 2(b)). Our results showed that LG's effect on cell proliferation was blocked when LG was chemically modified.

### 3.3. LG Was Transported into Cell via a Receptor-Mediated Pathway

**Figure 3:**
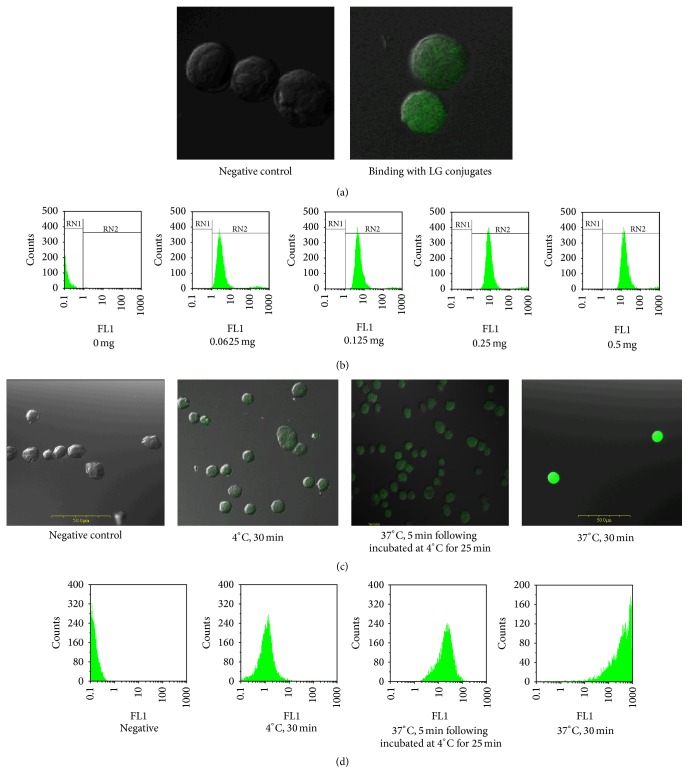
LG is transported into the cell cytoplasm after membranous LG-LG receptor binding process. (a) Immunofluorescence analysis by confocal microscopy (magnification = 1000x): FITC-LG (1 mg) was bound to cell membranes (green colored fluorescence) at 4°C after a 30 min incubation. (b) Flow cytometry: the cells were incubated with various doses of FITC-LG (0, 0.0625 mg, 0.125 mg, 0.25 mg, and 0.5 mg) at 37°C for 30 min and then their fluorescence intensities were assessed by flow cytometry. The results showed that, as the treatment dose increased, the fluorescence intensity detected in cells obviously increased (the peak shifts to the right or a higher intensity). (c) Immunofluorescence analysis by confocal microscopy: hybridoma cells were incubated using the following conditions: (1) negative control: no green fluorescence was observed in the cell membrane or the cytoplasm; (2) cells incubated with FITC-LG at 4°C for 30 min; green fluorescence was only observed on the cell membranes; (3) cells incubated with FITC-LG at 37°C for 5 min followed by an incubation at 4°C for 25 min: green fluorescence was observed in both the cell membrane and the cytoplasm; (4) cells incubated with FITC-LG at 37°C for 30 min: green fluorescence was observed mostly in the cytoplasm. (d) Flow cytometry further confirmed these results. The data indicated that FITC-LG could be transported into the cytoplasm after binding the cell membrane.

To verify the hypothesis that hybridoma cells possess membrane LG receptors that mediate the proliferation effect, we used immunofluorescence analysis to examine the binding of LG to its receptor on the cell membrane surface. The FITC-labeled LG was incubated with hybridoma cells and we observed FITC staining on the cell surface (Figure 3(a)). We applied the same procedure to analyze CHO cells; the FITC-labeled LG was not observed at the cell surface of CHO cells as it was on the hybridoma cells (data not shown). Interestingly, as the treatment dose of FITC-labeled LG increased, the fluorescence intensity detected from the hybridoma cells by flow cytometry also significantly increased (Figure 3(b)). Collectively, these data might indicate that hybridoma cells have specific receptors on the cell membrane that bind LG and then internalize it to mediate cell proliferation.

To test if LG transport into hybridoma cells is via a receptor-mediated pathway, we abated the metabolic activity of cells by decreasing the incubation temperature. FITC-labeled LG was incubated with hybridoma cells for different periods of time and at various temperatures. We then examined these cells for fluorescence using both confocal microscopy (Figure 3(c)) and flow cytometry (Figure 3(d)). When hybridoma cells were incubated with FITC-labeled LG at 4°C for 30 min, fluorescence was not observed on the cell surface or in the cytoplasm. When the hybridoma cells were first incubated with FITC-labeled LG at 37°C for 5 min and then incubated at 4°C for another 25 min, the fluorescence was expressed mainly on the cell membrane; in these cells, the FITC staining was of low intensity in cytoplasm. Interestingly, when the temperature was increased to 37°C for 30 min, the fluorescence was mainly present in the cytoplasm and the flow cytometry confirmed these results. These data might indicate that FITC-labeled LG is transported into the cell after binding to receptors in the plasma membrane.

### 3.4. Purification of Membranous LG Receptor from Hybridoma Cells

**Figure 4:**
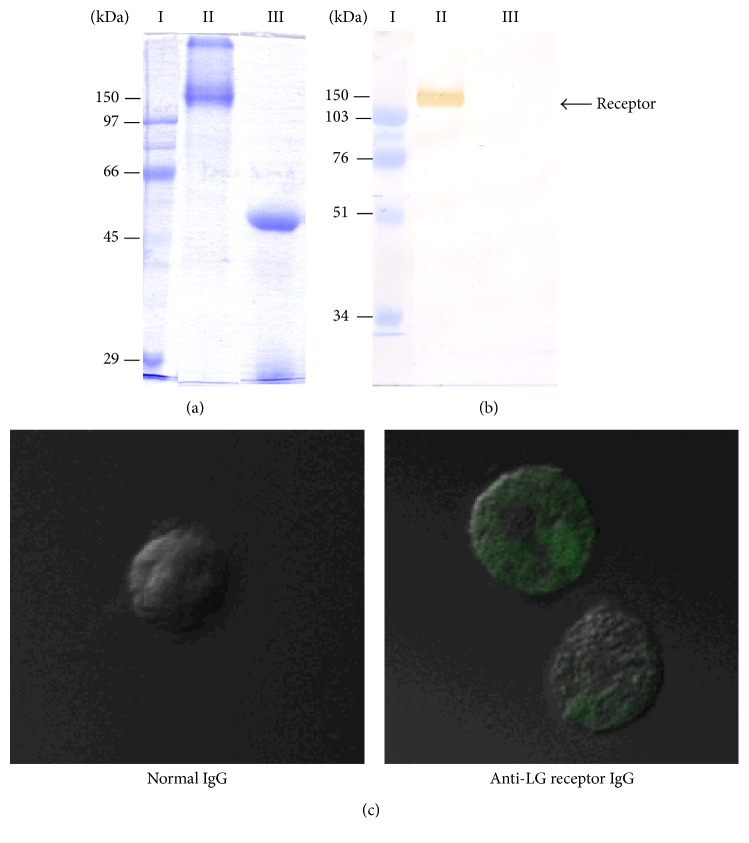
Purification of the LG receptor. (a) SDS-PAGE: lane I: protein ladder, lane II: LG receptor, and lane III: LG receptor with a reducing agent (*β*-ME); (b) Western blot. The receptor was purified using a LG affinity column. Samples from the column were analyzed by SDS-PAGE and Western blot analysis. A protein (later confirmed as the LG receptor) with a molecular weight of approximately 150 kDa was detected using a specific antibody. (c) Hybridoma cells were incubated with (1) IgG (1 mg) purified from normal rabbit serum (control) or (2) rabbit polyclonal antibodies specific to the LG receptor (1 mg). After labeling these two antibodies with FITC, the FITC-conjugated antibodies were incubated with the cells at 4°C for 30 min. We analyzed the localization of the FITC-labeled proteins using confocal microscopy (magnification = 1000x). Only rabbit polyclonal antibodies specific to the LG receptor (2) were detected on the cell membrane (green colored fluorescence). These data further confirmed that LG receptors were located on the surface of the membrane.

A LG affinity column was used to purify the receptor. After purification, the receptor was assessed by SDS-PAGE (Figure 4(a)) and Western blot (Figure 4(b)) analysis; we detected a protein with a molecular weight of approximately 150 kDa (Figure 4). Next, we examined this purified protein using MALDI-TOF MS and identified the protein as a membrane IgM.

To further assess the localization of the LG receptor, we immunized a rabbit with this purified protein and generated a polyclonal antibody that recognized the LG receptor. After conjugating this antibody to FITC, we analyzed the localization of the FITC-labeled proteins using confocal microscopy and found that the antibody was bound to the cell surface (Figure 4(c)). This evidence strongly implied that LG-binding receptors were located on the surface of the cell membrane.

**Figure 5:**
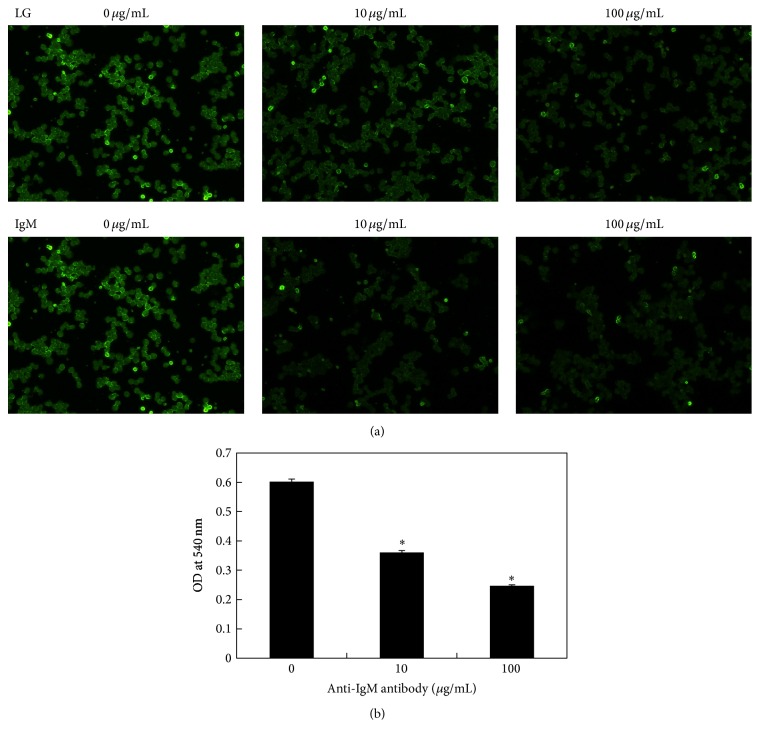
Effect of anti-IgM antibody on cell proliferation using competition assay. (a) Immunofluorescence analysis (magnification = 200x): various doses of anti-IgM (0, 10, and 100 *μ*g/mL) were added to samples containing 100 *μ*g/mL of FITC-labeled or unlabeled LG. We observed that anti-IgM antibody could compete with FITC-labeled LG for binding on the cell surface receptor; (b) MTT assay: samples from the receptor binding competition assay, LG progressively lost its ability to stimulate cell proliferation in a dose-dependent manner (^*∗*^ *p* < 0.001).

Finally, to confirm that the LG receptor is a membrane IgM, we used a fluorescence-based competition assay and the MTT assay. The data from the competition assay showed that the anti-IgM antibody competed with FITC-labeled LG to bind to the cell surface receptor (Figure 5(a)). Further, we observed a dose-dependent decrease in the cell proliferation effect (Figure 5(b)). This study further confirmed that the LG-induced cell proliferation was mediated via a LG-LG receptor binding process and that the LG receptor is a kind of membrane IgM.

## 4. Discussion

Whey protein is known to contain various biologically active ingredients which can potentially improve our health [28]. Patel highlighted that whey protein contains essential amino acids, bioactive peptides, antioxidants, and immunomodulators. These components of whey protein are important for radical scavenging, anti-inflammatory activities, antitumor effects, and immunostimulatory effects. These factors have been shown to regulate blood pressure, gut homeostasis, obesity, diabetes, muscle biosynthesis, bone structure, and protection from radioactivity [43]. Whey proteins are regarded as a potent substitute for serum in cell cultures. And although whey protein was shown to have various influences on human immune function via cell proliferation, there is little data regarding the role of individual proteins to these processes [30, 44, 45]. In our study, the dose-dependent treatment of LG positively correlated with cell proliferation when assessed by the MTT assay (Figure 1(a)). Further, we proved that LG is the major protein in bovine milk that stimulates cell proliferation by showing that LG-depleted milk did not stimulate cell proliferation (Figure 1(b)). LG comprises approximately 10–15% and 50% of total milk and whey proteins, respectively [1, 46–48]. Besides the promotion of cell proliferation, LG has been suggested to stimulate proinflammatory cytokine secretion and regulate the local Th1/Th2 response. IL-4, IL-10, IL-12, and TNF-*α* secretion have been measured in the growth medium after* in vitro* LG stimulation.* In vivo* rat immunization studies showed higher levels of IL-4 and INF-*γ* secretion [49]. Regarding the effect of LG on immune enhancement, LG may improve immune responses by promoting proinflammatory cytokine secretion and regulating the local Th1/Th2 balance. In this study, we first demonstrated that LG is directly responsible for cell proliferation.

We demonstrated that LG, but not HLG, promotes cell growth (Figure 1(a)). HLG has a different conformation than LG and is unable to promote cell proliferation (Figure 2(a)). Heat-induced structural changes to LG have been previously studies. At 65°C, reversible structural changes occur at a surface *β*-sheet (amino acids Lys8-Try19) and progress to an *α*-spiral (amino acids Thr125-Lys135). When LG is heated above 80°C, these conformational changes become irreversible modifications [49]. Our previous study clearly reported the effects of the thermal denaturation on purified LG in detail; various methodologies including circular dichroic spectroscopy, Western blot analysis, and both native- and SDS-PAGE were used to assess the structure of denatured LG. Thermally denatured LG undergoes severe conformational changes, including LG self-aggregation and LG conjugation to other milk proteins, when heated (70–80°C) [4]. Heat treatment of LG may result in a remarkable decrease in its ligand binding ability and its capacity for self-aggregation because intrachain disulfide bonds are disrupted [5, 8, 49]. Additionally, acetylation of LG replaces its lysine group with an acetyl group, which neutralizes lysine's positive charge. By disrupting the tertiary structure of LG by carboxymethylation, we find that this chemically altered LG loses its effect on cell proliferation (Figure 2(b)). Binding of LG to its receptor may occur because of the interaction between the positively charged lysine group of LG and a negatively charged residue on the receptor. Removal or decreasing the positive charge of a protein or peptide can result in loss of receptor binding [50]. Therefore, we suggest that the normal or functional LG conformation is important for ligand binding, receptor recognition, and cell growth stimulation.

Numerous studies have investigated LG transportation and several of them have suggested that LG enters cells through a cell surface receptor. Flower pointed out that LG increased the retinol uptake through the intestine of a mature rat, and intact LG could be detected in the child's blood two hours after ingesting milk [51]. This receptor-mediated transport mechanism is also utilized by low-density lipoprotein (LDL) and its receptor [52, 53], as well as by the megalin-like receptor for the uptake of various molecules [54]. Using immunofluorescence assays and flow cytometry, we demonstrated that LG enters cells through membranous LG-LG receptor (Figure 3). Further, we purified and identified the LG receptor as a membranous IgM (Figure 4). According to previous studies, enterocytes and M-cells are responsible for transporting LG from the intestinal lumen into the circulating system. LG is resistant to low pH which allows it to maintain its original structure and function after entering the circulation system [49]. Our immunofluorescence and MTT-based competition assay results again demonstrated that LG stimulated cell proliferation by binding to its receptor, membrane IgM (Figure 5). This LG receptor complex can initiate internalization and then promote cell growth. Interactions between these IgM receptors and their ligands can cause a series of biochemical responses, such as initiating gene transcription, anchoring a receptor to the cytoskeleton, receptor endocytosis, antigen presentation, cell differentiation, and cellular proliferation [55–57].

LG has also been investigated as a stable nanotransporter for covalently bound bioactive compounds [58]. Currently, developing an efficient method for drug delivery, such as nanoparticles, is crucial and urgent. It is proven that LG nanotransporters do not impair digestion; yet, it can successfully transport functional proteins and even enhance a compound's bioavailability [59, 60]. Our study directly demonstrated the mechanism of cellular LG transportation. Because LG has many useful features, elucidating the mechanism by which it is transported into a cell may aid in the development of protein-based nanoparticles.

## 5. Conclusions

In this study, we demonstrated that LG promotes cell proliferation, which directly proves that LG can enhance immune responses. We also showed that the conformation of LG was important for promoting cell proliferation. When LG was thermally or chemically denatured, it did not stimulate cell proliferation. Additionally, we showed that LG is the major protein in bovine milk that promotes cell proliferation through a receptor-mediated mechanism. The LG receptor was identified as membranous IgM. Taking all these data together, we conclude that properly folded LG enhances human immune responses by promoting cell proliferation via a receptor-mediated pathway. These results provide novel insights into the beneficial immune enhancing and modulating properties of dairy products. The regulatory mechanisms by which LG induces cell proliferation will be investigated in future studies.
